# Long-term changes in corneal densitometry and associated factors following small incision lenticule extraction for moderate and high myopia

**DOI:** 10.3389/fmed.2022.945894

**Published:** 2022-09-08

**Authors:** Changqin Xu, Dongmei Yang, Wuxiao Zhao, Zhigang Long, Zhe Zhang, Yang Shen, Xingtao Zhou, Jing Zhao

**Affiliations:** ^1^Eye Institute and Department of Ophthalmology, Eye and ENT Hospital, Fudan University, Shanghai, China; ^2^Key Laboratory of Myopia, NHC Key Laboratory of Myopia (Fudan University), Chinese Academy of Medical Sciences, Shanghai, China; ^3^Shanghai Research Center of Ophthalmology and Optometry, Shanghai, China; ^4^Department of Ophthalmology, The Third People's Hospital of Chengdu, The Affiliated Hospital of Southwest Jiaotong University, Chengdu, China; ^5^Department of Ophthalmology, Liuzhou Red Cross Hospital, Liuzhou, China

**Keywords:** corneal densitometry, myopia, small incision lenticule extraction, cornea, femtosecond laser

## Abstract

**Purpose:**

To investigate long-term trends in corneal densitometry and associated influencing factors following small incision lenticule extraction (SMILE).

**Methods:**

A comparative study was performed among 72 eyes of 38 patients undergoing SMILE. Eyes were divided into moderate myopia [mean spherical equivalent (SE),−4.22 ± 0.68D] and high myopia (mean SE,−7.63 ± 1.09 D) groups. Visual acuity, manifest refraction, corneal topography and corneal densitometry (CD) were evaluated preoperatively and 3 years postoperatively.

**Results:**

The efficacy indices at last postoperative follow-up (42.47 ± 0.51 months) were 0.92 ± 0.21 and 0.97 ± 0.22, the safety indices were 1.12 ± 0.17 and 1.14 ± 0.21 for high and moderate myopia, respectively (all *P* > 0.05). CD values in the 0–6 mm zone of the posterior corneal layer was statistically significantly lower than the preoperative values in both groups. Postoperative CD values (0–2 mm zone) in the posterior layer of the high myopia group were statistically significantly lower than in the moderate myopia group (*P* = 0.025); CD values (2–6 mm zone) in the anterior layer were higher in the high myopia group (*P* = 0.026). Correlation analyses showed that CD values in the 0–2 mm middle layer were negatively correlated with lenticule thickness in high myopia (*r* = −0.411, *P* = 0.016); there was a negative correlation between the CD values (0–6 mm) and corneal oblique trefoil in this group (*P* < 0.05).

**Conclusion:**

Corneal transparency in the 0–6 mm zone of the posterior cornea increased following SMILE in moderate and high myopia. Long-term CD values in high myopia may be correlated with oblique trefoil and lenticule thickness.

## Introduction

The cornea plays an important role in refractive medium of visual system. Corneal transparency is a key factor for achieving clear and bright vision. Postoperative corneal transparency directly determines the effects of refractive surgery and is one of the crucial evaluation indices for surgical safety. In clinical practice, corneal transparency is evaluated using both subjective and objective methodology. Subjective methodology mainly depends on evaluating corneal transparency *via* slit lamp microscopy. Objective methodology mainly includes measuring corneal densitometry (CD) using a Pentacam Scheimpflug camera to accurately evaluate corneal light backscatter and record sensitive alterations in corneal clarity (1).

Small incision lenticule extraction (SMILE) can correct refractive errors, including myopia and myopic astigmatism. SMILE is a minimally invasive corneal refractive that confirmed to be safe, effective, predictable, and stable (2–4). This procedure uses a femtosecond laser to scan the stroma layer of the cornea twice before manually removing the lenticule. According to previous studies, CD values increased significantly in the early postoperative stage following SMILE (5, 6) and progressively returned to the baseline (7, 8). That may explain why some patients experience temporary blurred vision and slow recovery following SMILE. Earlier studies have focused on comparing the changes in CD following SMILE and other refractive procedures (e.g., femtosecond laser-assisted LASIK) (9, 10). However, the characteristics of long-term CD postoperative trends following SMILE with different degrees of myopia remain unclear.

This study aimed to observe and compare long-term CD values among patients with moderate and high myopia following SMILE using Scheimpflug images. We likewise analyzed associated influencing factors, including preoperative spherical equivalents (SE), central corneal thickness (CCT), lenticule thickness (LT) and patient age. To our knowledge, this is the first study to assess long-term CD trends among patients with different degrees of myopia following SMILE.

## Patients and methods

### Patients and follow-up

In this comparative study, we evaluated 72 eyes among 38 participants with high myopia (34 eyes; 19 patients, 8 men, 11 women) and moderate myopia (38 eyes; 19 patients, 10 men, 9 women). We enrolled the current study population among patients undergoing refractive surgery for SMILE at the Fudan University Eye and ENT Hospital (Shanghai, China) from January to February 2018.

Inclusion criteria were as follows: (1) age ≥18 years; (2) stable refraction (< 0.50D increase within 2 years) without other ocular disorders; (3) preoperative refraction: high myopia group, SE < -6.0D, astigmatism <1.25D; moderate myopia group, sphere of myopia ranging from−3.0D to−6.0D, SE >-6.0D, astigmatism <1.25D; (4) residual bed thickness ≥280 μm. This study has been approved by the ethics committee of the Eye and ENT Hospital of Fudan University. Prior to participation, each patient voluntarily written an informed consent.

Each patient underwent preoperative and postoperative ophthalmological evaluations as follows: uncorrected distance vision acuity (UDVA), corrected distance visual acuity (CDVA), manifest refraction, corneal topography (Pentacam HR, Version 1.21r43, Oculus Optikgeräte GmbH, Wetzlar, Germany), axial length and intraocular pressure. The Pentacam scans were set to automatic mode. The CD values, wavefront aberrations, and CCT of the cornea were collected *via* one measurement with the image quality displayed with OK. Long-term postoperative follow-up was performed for patients for >3 years after surgery.

### Surgical procedures

SMILE procedures were performed by the same surgeon (XZ) using VisuMax femtosecond laser system (Carl Zeiss Meditec;Jena, Germany) with repetition rate of 500 kHz and pulse energy of 130 nJ. The lenticule diameter was set at 6.0–6.8 mm, the cap diameter of 7.5 mm at a depth of 120 μm (90° single-side cut, 2 mm length) and corneal cap thickness at 100–120 μm. This procedure has been described thoroughly in previous reports (2–4). After the surgery, each patient received standard topical treatment with antibiotics, steroids, and non-preserved artificial tear eye drops.

### Corneal densitometry measurement

Optical density images were collected to quantify CD values; the method was similar to that reported in previous literature (7). We evaluated corneal light backscatter expressed in grayscale units ranging from 0 (transparent) to 100 (opaque) measured from Scheimpflug images. CD values in each zone (0–2 mm, 2–6 mm, 0–6 mm) and each layer [anterior layer (the first 120 μm of the corneal thickness), central layer (between the anterior layer and the posterior layer), posterior layer (the last 60 μm of the cornea) and total layer] were recorded.

### Statistical analysis

Statistical analyses were performed using Statistical Package for the Social Sciences (statistical software version 25; SPSS Inc., Chicago, IL, United States). All values were expressed as means ± standard deviations. During the evaluation of high and moderate myopic groups, differences in age and sex at baseline were assessed using the two-sample *t*-tests and chi-square tests, respectively. The normality of distributions was assessed using the Shapiro—Wilk test. Mann–Whitney tests and independent-sample *t*-tests were performed for non–parametric and parametric comparisons as appropriate. The intervariable correlations were analyzed by Pearson or Spearman correlation coefficients. The mixed-effect linear model was adopted in multivariate analysis to avoid the effect of binocular data. *P* < 0.05 was considered statistically significant.

## Results

During our study, no intraoperative or postoperative complications occurred in any patient undergoing SMILE. Clinical parameters prior to and following SMILE are shown in Table 1.

**Table 1:** Demographics, preoperative and surgery data of high myopia vs. Moderate myopia groups.

**Characteristics**	**High myopia (*n* = 34 eyes)**	**Moderate myopia** **(*n* = 38 eyes)**	***P*** **value**
Age (mean ± SD, year) Range (Min, Max)	27.1± 4.6 (19, 36)	26.5 ± 4.3 (18, 36)	0.665
Sex (male/female)	8/11	10/9	0.516
Axial length (mm) Range (Min, Max)	26.63 ± 1.23 (24.50, 29.17)	25.70 ± 0.82 (23.71, 27.17)	0.000
Preoperative spherical error (D) Range (Min, Max)	−7.63 ± 1.09 (-9.75,−6.00)	−4.22 ± 0.68 (-5.50,−3.00)	0.000
Preoperative cylinder (D) Range (Min, Max)	−0.58 ± 0.37 (-1.25, 0.00)	−0.44 ± 0.25(-0.75, 0.00)	0.089
Preoperative SE (D) Range (Min, Max)	−7.92 ± 1.13 (-10.00,−6.38)	−4.44 ± 0.68 (-5.88,−3.25)	0.000
Preoperative CCT (μm) Range (Min, Max)	544.7 ± 27.4 (481, 589)	534.6 ±32.3 (477, 612)	0.160
LT (μm) Range (Min, Max)	142.6 ± 14.8 (104, 159)	100.3 ±12.5 (71, 123)	0.000
CDVA (logMAR) Range (Min, Max)	−0.11 ± 0.03 (-0.08, 0.00)	−0.03 ± 0.04 (-0.08, 0.00)	0.020

SE, spherical equivalent; CCT, central corneal thickness; LT, lenticule thickness; CDVA, corrected distance visual acuity; D, diopters.

### Refraction

#### Safety and efficacy

**Figure 1:**
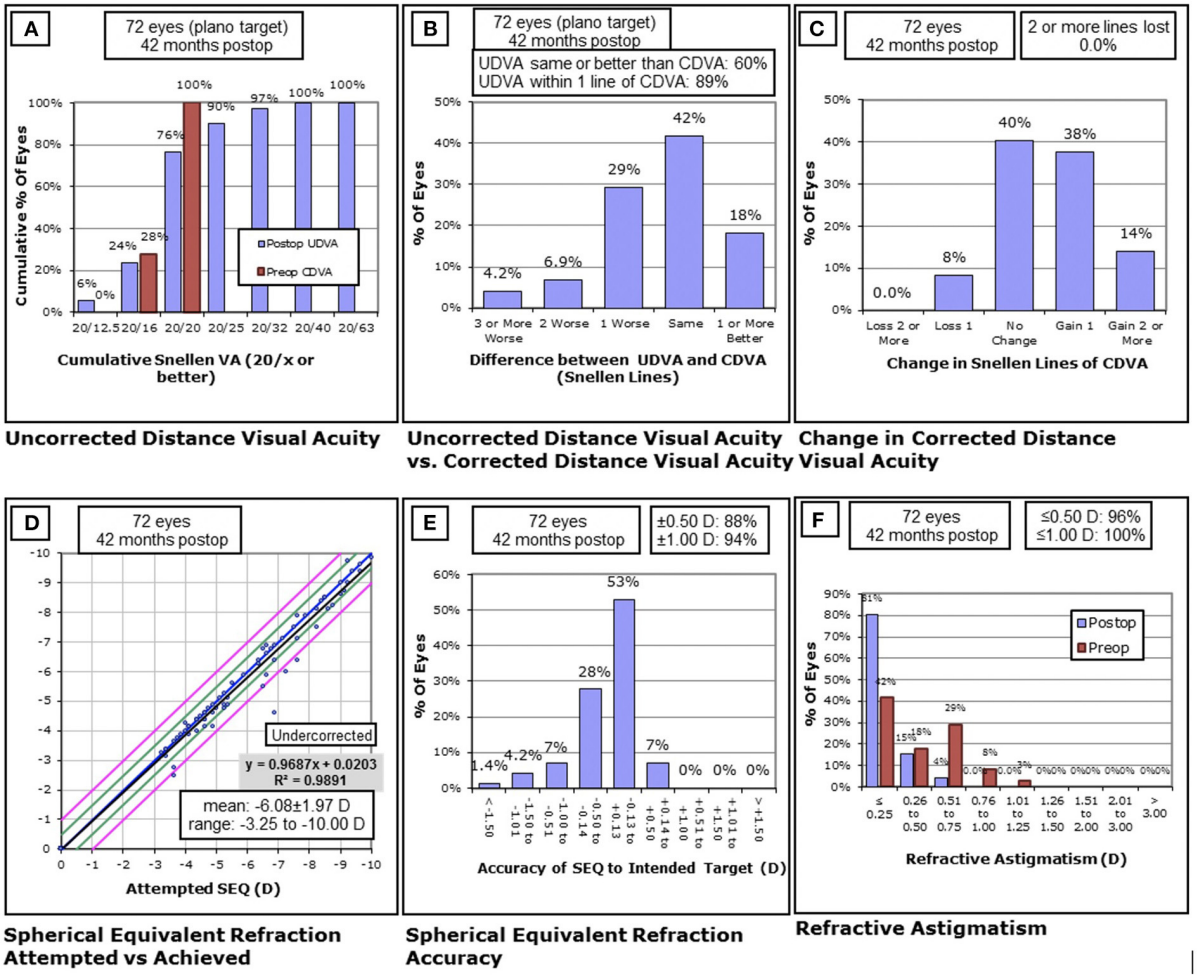
Clinical outcomes of 72 eyes with high and moderate myopia at the last follow-up after SMILE. **(A)** The postoperative uncorrected distance visual acuity (UDVA) vs. preoperative corrected distance visual acuity (CDVA); **(B)** the difference between preoperative CDVA and postoperative UDVA; **(C)** the change in CDVA; **(D)** the attempted vs. the achieved spherical equivalent refraction change; **(E)** the distribution of postoperative spherical equivalent refraction accuracy; **(F)** the postoperative residual astigmatism vs. preoperative astigmatism. D, diopters; Postop, postoperative; Preop, preoperative; mo, month.

The mean follow-up duration was 42.47 ± 0.51 months (range, 42–43 months). The safety (postoperative CDVA/preoperative CDVA) and efficacy indices (postoperative UDVA/preoperative CDVA) for all included eyes were 1.13 ± 0.19 and 0.95 ± 0.21, respectively; 76% of the patients (55 eyes) had a postoperative UDVA of ≥20/20 and 60% (43 eyes) of the patients had a postoperative UDVA greater than the preoperative CDVA. 8% of the patients (six eyes) lost lines in CDVA; 52% of the patients (37 eyes) gained one or more lines CDVA (Figures 1A–C).

The safety indices in the high and moderate myopia groups were 1.12 ± 0.17 and 1.14 ± 0.21, respectively, with no statistically significant intergroup differences (*P* = 0.676). The efficacy indices in the high and moderate myopia groups were 0.92 ± 0.21 and 0.97 ± 0.22, respectively, with no statistical differences between the two groups (*P* = 0.434). The postoperative SE values in the high myopia and moderate myopia groups were−0.28 ± 0.54D and−0.17 ± 0.29D, respectively, with no statistically significant intergroup differences (*P* = 0.488).

### Predictability

A total of 88% (63 eyes) of patients were within ±0.50D of the attempted refraction; 94% (68 eyes) were within ± 1.0D (Figures 1D,E). The plano target powers were −0.22 ± 0.42D (-2.25D; 0.50). Postoperative astigmatism was within 0.50D in 96% of patients (69 eyes). No eyes had postoperative astigmatism >1.0D (Figure 1F).

### CD values

In the moderate and high myopia group, the CD values at each of three zone (0–2, 2–6, and 0–6 mm) of the posterior corneal layer were decreased at 3.5 years postoperatively as compared to preoperative values (all *P* < 0.05). Besides, a significant decrease was observed in the CD values of the 0–2 mm zone of the posterior corneal layer (*P* < 0.001) in high myopia group than moderate myopia group; however, no statistically significant differences were detected in post-operative CD values in the anterior, middle, and whole layers as compared to preoperative values (Table 2).

**Table 2:** Preoperative and postoperative corneal densitometry in high and moderate myopia groups.

**CD(GSU)**	**High myopia**			**Moderate myopia**			**High vs. Moderate**	
	**Pre-op**	**Post-op**	***P*** **value**	**Pre-op**	**Post-op**	***P*** **value**	***P*** **value (Pre-op)**	***P*** **value (Post-op)**
AL 0–2 mm	22.3 ± 4.6	23.1 ± 0.9	0.288	23.1 ± 4.4	22.8 ± 1.8	0.365	0.697	0.968
AL 2–6 mm	20.1 ± 4.2	21.7 ± 0.8	0.166	20.7 ± 4.0	20.9 ± 1.6	0.635	0.888	0.026
AL 0–6 mm	20.4 ± 4.2	21.9 ± 0.8	0.171	21.0 ± 4.0	21.2 ± 1.6	0.613	0.933	0.052
CL 0–2 mm	13.1 ± 2.5	13.5 ± 0.9	0.347	13.6 ± 2.3	13.6 ± 0.9	0.425	0.538	0.334
CL 2–6 mm	11.8 ± 2.2	12.7 ± 0.4	0.161	12.2 ± 2.0	12.4 ± 0.7	0.771	0.709	0.213
CL 0–6 mm	11.9 ± 2.2	12.8 ± 0.5	0.161	12.3 ± 2.0	12.6 ± 0.7	0.667	0.655	0.445
PL 0–2 mm	11.3 ± 1.9	9.5 ± 0.8	0.000	11.6 ± 1.8	9.9 ± 0.7	0.000	0.631	0.025
PL 2–6 mm	10.3 ± 1.6	9.7 ± 0.7	0.046	10.5 ± 1.6	9.6 ± 0.5	0.001	0.615	0.827
PL 0–6 mm	10.4 ± 1.7	9.7 ± 0.7	0.017	10.7 ± 1.6	9.7 ± 0.5	0.001	0.627	0.870
TL 0–2 mm	15.6 ± 2.9	15.3 ± 0.7	0.952	16.1 ± 2.8	15.4 ± 1.0	0.231	0.892	0.296
TL 2–6 mm	14.0 ± 2.6	14.7 ± 0.5	0.255	14.5 ± 2.5	14.3 ± 0.8	0.429	0.843	0.107
TL 0–6 mm	14.2 ± 2.7	14.7 ± 0.5	0.255	14.7 ± 2.5	14.5 ± 0.8	0.400	0.865	0.218

AL, anterior layer; CL, central layer; PL, posterior layer; TL, total layer; Preop, preoperative; Postop, postoperative.

No statistically significant differences were observed in preoperative CD values between the two groups. The postoperative CD values in the 0–2 mm zone of the posterior corneal layer in the high myopia group were statistically significantly lower than in the moderate myopia group (*P* = 0.025), whereas the CD values in the 2–6 mm zone of the anterior corneal layer in the high myopia group were statistically significantly higher than that in the moderate myopia group (*P* = 0.026). There were no statistically significant intergroup differences in the other areas (Table 2).

### Corneal wavefront aberrations

There were no statistically significant intergroup differences for higher order aberrations (HOAs) prior to surgery (Table 3). Postoperative long-term HOAs were 1.15 ± 0.33 μm in high myopia and 0.63 ± 0.13 μm in moderate myopia, with statistically significant intergroup differences (*P* < 0.001). Vertical coma (-0.65 ± 0.50 μm) and spherical aberration (0.50 ± 0.19 μm) in the high myopia group were statistically significantly higher than the respective values (-0.32 ± 0.20 μm, 0.28 ± 0.10 μm) in the moderate myopia group (*P* = 0.001); however, there were no statistically significant intergroup differences in horizontal trefoil, oblique trefoil, or horizontal coma (Table 3).

**Table 3:** Preoperative and last postoperative follow-up corneal wavefront aberrations in high and moderate myopia.

**Aberration comonents(um)**	**Pre-op**			**Post-op year 3.5**		
	**High myopia**	**Moderate myopia**	***P*** **value**	**High myopia**	**Moderate myopia**	***P*** **value**
HOAs	0.25 ± 0.11	0.25 ± 0.11	0.919	1.15 ± 0.33^*^	0.63 ± 0.13^*^	0.000
Oblique trefoil	−0.06 ± 0.08	−0.07 ± 0.11	0.660	−0.11 ± 0.13	−0.08 ± 0.11	0.301
Vertical coma	−0.01 ± 0.10	−0.02 ± 0.09	0.617	−0.65 ± 0.50^*^	−0.32 ± 0.20^*^	0.000
Horizontal Coma	0.03 ± 0.11	0.01 ± 0.11	0.472	0.27 ± 0.54	0.10 ± 0.30	0.109
Horizontal trefoil	−0.01 ± 0.08	0.01 ± 0.08	0.108	−0.02 ± 0.10	−0.01 ± 0.11	0.710
Spherical	0.11 ± 0.10	0.10 ± 0.06	0.888	0.50 ± 0.19^*^	0.28 ± 0.10^*^	0.000

HOAs, higher order aberrations; Preop, preoperative; Postop, postoperative.

* p <0.05 High myopia vs. Moderate myopia.

### CD-associated factors

In the high myopia group, CD values in the 0–2 mm zone of the middle corneal layer were negatively correlated with LT (*r* = −0.411, *P* = 0.016), and CD values in the 0–6 mm zone in all corneal layers were not correlated with age or with the preoperative SE (*P* > 0.05) (Table 4). In the moderate myopia group, CD values in the range of 0–6 mm in all corneal layers were not correlated with age, LT, or the preoperative SE (Table 4).

**Table 4:** Spearman correlation between corneal densitometry at last follow-up and preoperative parameters in high and moderate myopia group.

**Pre-op**	**High myopia**						**Moderate myopia**					
	**Age**		**LT**		**SE**		**Age**		**LT**		**SE**	
	***R***	***P*** **value**	***R***	***P*** **value**	***R***	***P*** **value**	***R***	***P*** **value**	***R***	***P*** **value**	***R***	***P*** **value**
AL 0–2 mm	−0.315	0.070	−0.270	0.122	0.134	0.449	−0.120	0.474	0.120	0.474	−0.119	0.476
AL 2–6 mm	0.002	0.993	−0.161	0.363	0.192	0.275	−0.032	0.847	0.188	0.259	−0.176	0.290
AL 0–6 mm	−0.042	0.815	−0.187	0.291	0.201	0.255	−0.046	0.786	0.186	0.263	−0.179	0.282
CL 0–2 mm	0.031	0.860	-.411^*^	0.016	0.296	0.089	−0.171	0.305	0.226	0.172	−0.216	0.192
CL 2–6 mm	0.140	0.429	−0.254	0.147	0.263	0.112	0.036	0.832	0.083	0.618	−0.074	0.657
CL 0–6 mm	0.112	0.527	0.108	0.108	0.276	0.114	−0.021	0.900	0.094	0.573	−0.090	0.592
PL 0–2 mm	−0.025	0.888	−0.082	0.644	0.151	0.395	−0.141	0.397	−0.101	0.545	0.076	0.648
PL 2-6 mm	0.133	0.453	0.089	0.618	0.074	0.677	0.093	0.577	−0.201	0.227	0.171	0.305
PL 0–6 mm	0.121	0.496	0.078	0.660	0.077	0.664	0.065	0.700	−0.202	0.225	0.175	0.293
TL 0–2 mm	−0.067	0.706	−0.338	0.051	0.278	0.111	−0.154	0.355	0.074	0.658	−0.085	0.614
TL 2–6 mm	0.153	0.388	−0.156	0.379	0.271	0.121	0.020	0.907	0.091	0.585	−0.089	0.597
TL 0–6 mm	0.121	0.496	0.078	0.660	0.077	0.664	0.003	0.987	0.087	0.603	−0.090	0.591

AL, anterior layer; CL, central layer; PL, posterior layer; TL, total layer; LT, lenticule thickness; SE, Spherical Equivalent; R, correlation coefficient.

* *p* < 0.05.

Correlation analyses with respect to postoperative CD values and corneal wavefront aberrations showed no statistically significant correlations when comparing these in each corneal layer in the moderate myopia group. In the high myopia group, we observed negative correlations between CD values in the whole layer (0–6 mm) zone and oblique trefoil (*P* < 0.05) (Table 5).

**Table 5:** Correlation between total corneal densitometry (0-6 mm annulus) and corneal wavefront aberrations at last follow-up.

**CD(TL 0–6mm)**		**HOAs**	**Oblique trefoil**	**Vertical coma**	**Horizontal coma**	**Horizontal trefoil**	**Spherical**
High myopia	R	−0.157	−0.347	0.019	−0.222	−0.208	−0.136
	*P* value	0.376	0.044	0.916	0.206	0.237	0.443
Moderate myopia	R	0.092	−0.219	0.057	−0.075	0.050	−0.138
	*P* value	0.584	0.187	0.734	0.654	0.768	0.408

CD, corneal densitometry; HOAs, higher order aberrations; R, correlation coefficient.

## Discussion

CD measurement based on the Scheimpflug imaging system is an objective modality for assessing corneal transparency (11). Compared with other measurements, this methodology allows for rapid assessment of backscattering across the cornea; it is gradually being applied to monitor corneal surgical outcomes, including corneal collagen crosslinking (12), keratoplasty (13, 14), and corneal refractive surgery. Several studies have reported alterations in CD following SMILE (7, 8). What is more, it has been reported that most CD values increased significantly in the early postoperative period compared with preoperative values and gradually returned to baseline following SMILE (9, 15). However, the characteristics of long-term postoperative CD trends, given the different degrees of myopia, remain unclear. In the present study, we aimed to assess long-term CD trends and alterations among patients with moderate and high myopia following SMILE. Additionally, we focused on CD values in the 0–6 mm circular zone of the pupil region, as the corneal opacity in this zone is most likely to affect visual quality.

In long-term follow-up, we found that CD values in the posterior corneal layer were statistically significantly decreased in both groups as compared with preoperative values, signifying that the transparency of the posterior layer of the cornea in both groups could be better-quality. This increase in the CD values of posterior layer may be related to the quality of images obtained from the posterior layer of the cornea. Li et al. (16) showed that corneal cell density and basal nerve density were lower following SMILE than preoperatively *via in vivo* confocal microscopy (IVCM); there were no signs of recovery during the follow-up period. Therefore, we believed that the thinning of stromal thickness would result in the reduction of posterior reflective particles (i.e., corneal cells, collagen fibers), which might make the assessment of posterior backscattering more accurate. Additionally, we found that the CD values in the 2 mm zone within the posterior layer in the high myopia group were statistically significantly lower than that in moderate myopia (*P* = 0.025), indicating that the LT was directly proportional to the CD of the posterior corneal layer. With respect to long-term follow-up, the postoperative CD values for the posterior corneal layer in both groups were statistically significantly reduced as compared with preoperative values. We thus speculate that the transparency of the posterior corneal layer in both groups was improved.

The study indicated that there was no statistically significant difference in CD values in the 0–6 mm zone of the anterior and intermediate layers in both groups, suggesting that femtosecond laser damage to the cornea was slight and that SMILE had little effect on the CD values of the anterior and intermediate cornea. During long-term follow-up, the CD values of the anterior and intermediate corneal layers in the 0–6 mm zone was stable in both groups and there were no statistically significant alterations when compared preoperatively. This result differs from that of the study conducted by Han et al. (9) who found that CD values statistically significantly decreased in the three layers and the three zones of the SMILE group at the three-year follow-up visit as compared with the preoperative values. We considered that the differing changes in the CD values between the two studies might be due to the differences in the samples included within the respective studies. Our study enrolled two groups with myopia diopters ranging from−6.00D to−9.75D and−3.00D to−5.50D, respectively, the average age of the patients included in our investigation was <30 years (27.1 ± 4.6 years in the high myopia group and 26.5 ± 4.3 years in the moderate myopia group). However, in Han's study, the range of diopters included in the SMILE group was not specified, the average age of the patients was >30 years (30.47 years). According to previous studies, corneal densitometry increases with age (17) and light backscatter was lower in the high myopia cornea than in the normal cornea (18).

The postoperative CD values in the 2–6 mm anterior layer was statistically significantly higher in the high myopia group when compared with the moderate myopia group, which was assumed to be associated with corneal epithelial remodeling. Earlier studies have found that the eccentricity of the central corneal epithelial thickness (0–6 mm) increased following SMILE and that the quantity of corneal epithelial thickening was positively correlated with the extent of the myopia diopter to be corrected (19). The most obvious thickening was detected in the para-central zone (2–5 mm) (20). Therefore, we speculated that, as compared with moderate myopia, the increase in the thickness of the para-central corneal epithelium in patients with high myopia was more obvious. This may be the main reason mediating the observed differences in CD values in the 2–6 mm anterior layer when comparing the two groups.

Correlation analysis have shown that CD values in the 0–2 mm zone of the middle layer were negatively correlated with LT (*r* = −0.411, *P* = 0.016) in high myopia, indicating that increase in the thickness of the lenticule might be results in lower CD values. However, there was no statistically significant correlations between CD values in the 2–6 mm zone of the middle layer and the LT. We speculate that this result is mainly due to a combination of the following two factors. First, the surgically removed lenticule is convex and the center of the lenticule is statistically significantly thicker than the periphery. Thus, the higher the myopia diopter, the greater the change in the central thickness of the cornea. Second, previous reports indicated that, as compared with the periphery, corneal collagen fibers in the anterior central portion of the pupil are packed more closely (21) and that the CD values in the 0–2 mm zone are larger than in the 0–6 mm zone (22). Therefore, compared with other zones, a femtosecond laser with the same amount of energy will have a more obvious effect on collagen fibers in the 0–2 mm zone of the central pupil, and the alterations in terms of CD values in this zone will likewise be obvious. However, this statistically significant correlation was not observed in the moderate myopia group. The reason for this finding may be due to the fact that the lenticule has a certain threshold thickness. In our study, the LT of the high myopia group was 142.6 ± 14.8 μm, which was statistically significantly higher than in the moderate myopia group (100.3 ± 12.5 μm; *P* < 0.001). However, this hypothesis needs to be tested in future studies.

According to previous studies, CD values are statistically significantly correlated with patient age (16, 23). However, we found that there were no statistically significant correlations between CD values of each layer of the 0–6 mm zones with respect to patient age (*P* > 0.05). The potential reason for this finding is that the average age of the patients included in our investigation was <30 years (27.1 ± 4.6 years in the high myopia group and 26.5 ± 4.3 years in the moderate myopia group) and the average postoperative observation period was 3.5 years, which might exclude the influence effect of aging on CD values. However, the likelihood that postoperative long-term CD values differ with age warrants additional study.

To the best of our knowledge, there are few studies evaluating whether long-term trends in CD values following SMILE affect corneal aberrations. Our study showed that a negative correlation between CD values in the whole corneal layer in the 0–6 mm zone and the corneal oblique trefoil following surgery (*P* < 0.05). However, Han et al. (9) demonstrated no correlation between CD values and alterations in corneal aberration. This might be due to differences in study objectives. These results warrant the need for additional study within high-powered investigations.

The study has certain limitations. First, the sample size was relatively small. Second, there was no intermediate follow up period between pre and 2 years post operation. Further studies with larger sample sizes are essential to explore relationships between CD values following SMILE and increased follow-up time points.

In conclusion, our findings demonstrate that, as compared with the preoperative values, CD values decreased in the posterior corneal layer among patients with high and moderate myopia following SMILE, while the CD values in the anterior and middle corneal layers remained unchanged. These findings are likely mediated by the fact that long-term CD values in the high myopia group may be correlated with LT and corneal oblique trefoil.

## Data availability statement

The original contributions presented in the study are included in the article/Supplementary material, further inquiries can be directed to the corresponding authors.

## Ethics statement

The studies involving human participants were reviewed and approved by the Ethics Committee of the Eye and ENT Hospital of Fudan University. The patients/participants provided their written informed consent to participate in this study.

## Author contributions

Study concept and design: CX, XZ, and JZ. Data collection: CX,WZ, ZL, and XZ. Analysis and interpretation of data: CX, DY, ZZ, YS, and JZ. Writing the manuscript: CX, DY, and JZ. Critical revision of the manuscript, Obtaining funding and supervision: JZ and XZ. All authors contributed to the article and approved the submitted version.

## Funding

Supported by the Project of Shanghai Science and Technology (Grant No.20410710100) (Grant No. 21Y11909800), Clinical Research Plan of SHDC (SHDC2020CR1043B),the Project of Shanghai Xuhui District Science and Technology (XHLHGG202104), Shanghai Engineering Research Center of Laser and Autostereoscopic 3D for Vision Care (20DZ2255000),Construction of a 3D digital intelligent prevention and control platform for the whole life cycle of highly myopic patients in the Yangtze River Delta (21002411600).

## Conflict of interest

The authors declare that the research was conducted in the absence of any commercial or financial relationships that could be construed as a potential conflict of interest.

## Publisher's note

All claims expressed in this article are solely those of the authors and do not necessarily represent those of their affiliated organizations, or those of the publisher, the editors and the reviewers. Any product that may be evaluated in this article, or claim that may be made by its manufacturer, is not guaranteed or endorsed by the publisher.
